# Randomized comparative field clinical trial of hydrocortisone aceponate/gentamicin/miconazole otic suspension vs. mometasone furoate/florfenicol/terbinafine otic solution for the treatment of canine otitis externa

**DOI:** 10.3389/fvets.2026.1901247

**Published:** 2026-10-28

**Authors:** J. Griffin, C. von Simson, L. Poincelot, P. Jasmin

**Affiliations:** 1 Animal Dermatology Clinic, Louisville, KY, United States; 2 Virbac Corporation, Westlake, TX, United States; 3 Virbac S.A., Carros, France

**Keywords:** canine otitis externa, dog, efficacy, otic product, OTIS3, treatment

## Abstract

This randomized, masked, multi-center field clinical trial compared two otic formulations: a gentamicin, miconazole, and hydrocortisone aceponate otic suspension (Easotic™, Virbac) administered once daily for five days, and a single-dose florfenicol, terbinafine, and mometasone furoate otic solution (Claro™, Elanco). Eighty-six client-owned dogs with acute otitis externa (OTIS3 score ≥ 5) were evaluated at 4 h post-application and on Days 2, 7, and 28. Based on the percentage of dogs achieving an OTIS3 score ≤ 3 at Day 28, both treatments (Easotic™ vs. Claro™) showed high efficacy (Intention-to-Treat (ITT): 85% vs. 79%, *p* = 0.400; Per Protocol (PP): 94% vs. 88%, *p* = 0.261). Easotic™ demonstrated a higher percentage of OTIS3 score reduction compared to Claro™ from Day 2. At Day 2, Easotic™ achieved greater improvements in ear discharge, odor, dirtiness, and stenosis compared to Claro™. Veterinarians' overall assessment of “*Excellent*” response was higher for Easotic™ at Day 7 (ITT: 50% vs. 26%, *p* = 0.009; PP: 49% vs. 29%, *p* = 0.010) and Day 28 (ITT: 62% vs. 36%, *p* = 0.017; PP: 76% vs. 44%, *p* = 0.002;). Similarly, 91% of the owners assessed the response to treatment as “*Good*” or “*Excellent*” with Easotic™, and 89% with Claro™. Notwithstanding the differences in dosing regimens, both products received comparable evaluations from owners regarding their physical and functional characteristics. The results of this study confirm the efficacy of Easotic™ in the management of otitis externa, notably to reduce ear discharge, stenosis, odor and dirtiness from Day 2.

## Introduction (background and objectives)

Canine otitis externa (OE) is a widespread, frequently recurrent inflammatory disorder of the external ear canal. Clinical remission includes managing its multifactorial etiology via the primary–secondary–predisposing–perpetuating framework (1). Primary factors initiate the disease process; primary inflammatory disorders are the most common drivers, alongside foreign bodies, parasites, endocrinopathies, and neoplasia. Predisposing factors alter the ear canal microenvironment to facilitate disease development and progression. Primary inflammation enables secondary complications, predominantly driven by local dysbiosis rather than novel pathogen acquisition. In erythemato-ceruminous otitis externa (ECOE), this dysbiosis typically manifests as yeast or bacterial overgrowth (2). Unresolved primary and secondary factors lead to perpetuating pathological changes, beginning as epidermal and ceruminous gland hyperplasia and advancing to severe stenosis, occlusion and fibrosis (1, 3–5). Clinical signs include pain, discharge, and malodor (5–7). Successful management requires anti-inflammatory therapy combined with targeted antimicrobial and antifungal treatment to restore ear canal homeostasis (2, 8).

Several commercial otic products are available with different combinations of glucocorticoids and antimicrobials in varying dosages and/or modes of administration (1, 3).

Choosing a product that resolves the pain and inflammation and is easy to administer is necessary to provide fast improvement and prevent chronic changes from occurring to the ear canal (9–11). This study evaluated the comparative efficacy of two veterinary products, a gentamicin, miconazole, and hydrocortisone aceponate otic suspension (Easotic™, Virbac) and a florfenicol, terbinafine, and mometasone furoate otic solution (Claro™, Elanco) for the treatment of acute canine otitis externa.

## Materials, animals and methods

This study was a multi-center, randomized, parallel-group, masked clinical field trial.

### Ethics

The design of this study was approved by the Virbac Corporation Ethical Review Committee. All dogs were recruited within the veterinarians' clinics. Participation in the clinical trial was based on the voluntary intention of the owners informed orally and in writing about the content of the clinical trial and their rights.

### Animals

The dogs enrolled in the study were client-owned dogs visiting the veterinary clinics located in the United States. Written informed consent was obtained from all dog owners prior to performing baseline screening procedures and treatment randomization. Dogs enrolled were 7 months of age or older, of any breed or sex, and neutered or intact. The dogs were diagnosed with clinical signs of an acute episode of non-parasitic otitis externa with a minimum OTIS3 (Otitis Index Score) score of 5 (12) and cytological evidence of the presence of rods, cocci or yeast. Dogs were in good general health or with stable chronic conditions. In cases of bilateral otitis, both ears were treated with the same product, but only the ear with the highest OTIS3 score was selected for evaluation during this trial. Acute otitis externa was defined as a clinical sign duration of less than 1 month (6). The exclusion criteria consisted of a ruptured tympanic membrane, purulent exudate, otic ectoparasites, or a foreign body identified during the initial otoscopic examination. No sedation was required. Dogs presenting with advanced proliferative or occlusive otitis and ear canal neoplasia were excluded. Pregnant or lactating dogs or dogs with any concomitant disease that could interfere with the evaluation of the treatment were not included. Dogs were excluded if they had received short-acting corticosteroids, NSAIDs, antibiotics, or antifungals within 14 days; long-acting corticosteroids within 2 months prior to enrollment. Concomitant allergen-specific immunotherapy (>12 months duration; stable ≥6 months) and systemic immunomodulators (cyclosporine, oclacitinib, or lokivetmab for >2 months at stable doses) were permitted provided dosing remained unchanged throughout the study period. Dogs with recurrent otitis externa were eligible for inclusion; however, those treated with otic formulations containing hydrocortisone aceponate / gentamicin sulfate / miconazole nitrate or mometasone furoate / florfenicol / terbinafine within 3 months prior to enrollment were excluded.

Detailed diagnostic workups for primary or predisposing factors fell outside the study scope. Any additional clinical management, including further diagnostics or procedures, was conducted at the discretion and sole responsibility of the attending veterinarian and owner.

All client-owned dogs whose owners consented and who met all baseline inclusion criteria and received at least one dose of study treatment were retained in the primary Intention-to-Treat (ITT) population.

Post-randomization, dogs whose baseline cytological examination revealed exclusively rod-shaped bacteria were excluded from the Per Protocol population of the Claro group. This provided a label-aligned evaluation while preserving a representative baseline and reflecting real-world veterinary practice. The Per Protocol population was evaluated secondary as an exploratory sensitivity analysis.

### Treatment

At Day 0, enrolled dogs were randomly allocated in a 1:1 ratio to receive either Easotic™ or Claro™. Sequential case identification numbers were assigned at each clinic in chronological order of subject presentation. Randomization was executed across seven clinical study sites using a centralized electronic data capture system (Vision™, Prelude Dynamics, Austin, TX, USA). The system implemented a single global, unstratified, “just-in-time” algorithm that dynamically calculated treatment allocations at the precise moment of subject entry, without site-level block constraints or *post-hoc* re-balancing. This dynamic process generated a random treatment distribution equivalent to a single global pre-generated allocation schedule while maintaining strict allocation concealment to minimize selection bias and preserve investigator masking.

Veterinarian masking was maintained through strict separation of roles at each study site. Examining veterinarians performed all clinical evaluations while remaining blinded to treatment assignment, dispensing, and drug accountability. A designated unblinded dispenser at each veterinary clinic managed drug allocation and administration. On Day 0, prior to ear product administration, a clap test was performed and ear swabs were collected for semi-quantitative cytology and submission to the central laboratory. Before any product administration, the external ear canal of the affected ear was cleaned with sterile saline by the dispenser. No further ear cleansing was allowed during the entirety of the study.

One group of dogs received Easotic^TM^ Otic suspension for dogs (hydrocortisone aceponate, gentamicin sulfate, miconazole nitrate, Virbac Corporation). The initial dose was administered by the dispenser, and the subsequent four doses were administered by the owners at home according to the instructions provided by the dispenser. On Day 7, the dispenser assessed owner compliance. Owner compliance was evaluated by the dispenser, who conducted a structured owner interview and audited the compliance form completed after each treatment administration. The bottle was weighed on Day 0 prior to initial administration and then on Day 7. To account for minor scale variation while maintaining strict dosing compliance, a ±10% mass variance threshold was applied to define acceptable owner dosing adherence across the four home administrations.

The other group received Claro^TM^ Otic solution (mometasone furoate, florfenicol, terbinafine, Elanco US Inc). A single 1 mL dose was administered by the dispenser.

Both products were administered in accordance with the respective manufacturer's recommendations.

### Monitoring

#### Veterinarian assessment

Dogs were monitored across a 28-day study period. At baseline (Day 0), the veterinarian assessed enrollment criteria, requiring an OTIS3 score ≥ 5 and microscopic confirmation of rods, cocci or yeast organisms. On Day 0, prior to the ear product administration, a clap test was performed and ear swabs were collected. The veterinarian performed the semi-quantitative cytology. Following initial treatment administration on Day 0, veterinary evaluations were performed at four post-baseline time points: 4 h post-application, and on Days 2, 7, and 28. Prior to trial initiation, examining veterinarians at all clinical study sites received standardized training on the OTIS3 scoring guidelines and study protocol procedures to standardize clinical evaluations. To ensure longitudinal consistency and eliminate interobserver variation within cases, each dog was evaluated by the same blinded examining veterinarian at every scheduled study visit. Clinical severity of otitis externa was evaluated using the OTIS3 scoring system, adapted from the canine otitis externa clinical scoring index described by Nuttall and Bensignor (12). The OTIS3 index evaluates four distinct clinical parameters: erythema, edema, exudate (discharge), and ulceration (erosion), each graded on a 4-point scale (0 = Absent; 1 = Mild; 2 = Moderate; 3 = Severe), with the final OTIS3 score representing the cumulative sum of these individual values (0–12). Additionally, four secondary ear characteristics (stenosis, odor, dirtiness, pain) were scored using a 0-3 scale (12).

On Days 7 and 28, the veterinarian performed a subjective global assessment of response to treatment. For each dog, the response to treatment was based on the subjective evolution of clinical signs relative to the initial examination (Day 0), using a 4-point ordinal scale (0 = Poor: no change/worsening; 1 = Moderate: slight improvement; 2 = Good: clear improvement; 3 = Excellent: complete resolution). To prevent bias, veterinarians performed assessments without disclosing or sharing results with dog owners.

The treatment success was defined as the decrease of the OTIS3 score from ≥5 to ≤ 3. The treatment success was recorded at Day 7 and Day 28.

At Day 7, if the OTIS3 score was ≥ 4, the case was considered a failure, and the dog was withdrawn from the study and treated appropriately based on a mutual medical decision between the veterinarian and the owner. Subsequent clinical management, including diagnostic investigations, complementary procedures, or rescue treatments, was provided at the discretion of the attending veterinarian in consultation with the owner. All interventions conducted outside the study protocol were strictly the responsibility of the veterinarian and dog owner.

#### Owner assessment

Owners performed subjective assessments of physical manifestations. Parameters including pain, pruritus, odor, and dirtiness were quantified using a validated 10 cm visual analog scale (VAS) at baseline (Day 0), 4 h post-application on Day 0, and subsequently on Days 2, 7, and 28 (13). The VAS consisted of a line bounded by vertical indicators at each end, anchored with “Absent” (0 cm) at the left boundary and “Severe” (10 cm) at the right boundary. Owners placed a single vertical mark along the line corresponding to the perceived severity of the manifestation. Measurements were recorded in millimeters from the 0 cm line to the owner's mark, generating a continuous score ranging from 0 to 10 cm without scale transformation.

On Day 7, owners provided a subjective global assessment of the response to treatment (based on improvement of observed manifestations). Concurrently, specific product characteristics (texture, cosmesis on the ear, and odor) were evaluated. These parameters were rated on a four-point scale (Excellent, Good, Moderate, or Poor).

#### Cytological assessment

An ear swab was performed on Day 0, Day 7 and Day 28. All cytological evaluations were performed by a masked veterinarian who was blinded to treatment assignments. Ear canal exudate was collected from the junction of the vertical and horizontal ear canals using a sterile cotton swab and gently rolled onto a clean glass slide to prepare a thin smear. Slides were air-dried at room temperature and stained with a modified Wright–Giemsa stain by performing 5–10 sequential 1-second dips per reagent. Stained slides were rinsed with tap water, thoroughly air-dried, and examined under light microscopy at times 1,000x total magnification (oil immersion). Cytological assessment was performed using the validated Budach and Mueller scale (14). This 5-point grading system (0 to 4+) was employed to semi-quantitatively evaluate the presence and density of 3 categories: bacteria (rods and cocci), yeast, and inflammatory cells within the cytological preparations. Each category is assigned a grade on a scale from 0 to 4+ based on the quantity of the elements and how easily they can be found on the cytological preparations. A score of 0 indicates that no bacteria, yeast, or inflammatory cells are present. A score of 1+ is given when there are occasional elements present, but the examiner must scan the slide carefully to detect them. A score of 2+ indicates that elements are present in low numbers but can be found without difficulty. A score of 3+ is assigned when there are larger numbers present that are rapidly detectable. Finally, a score of 4+ represents massive amounts of bacteria, yeast, or inflammatory cells that are easily detectable. The total cytological score is calculated by summing the individual grades assigned to each evaluated category. The type of microorganisms identified was specified by documenting the presence or absence of cocci, rods, or yeast.

On Day 0, an additional ear swab was collected and sent to a central laboratory (IDEXX BioAnalytics, West Sacramento, CA 95605) for culture and susceptibility testing. Samples were processed using standard veterinary diagnostic protocols, incorporating aerobic culture on selective and non-selective media, including blood and MacConkey agars for bacterial pathogens, and Sabouraud dextrose agar for yeast isolation, incubated under conventional laboratory conditions. Antimicrobial susceptibility testing was performed via the agar disk diffusion (Kirby-Bauer) method, with interpretive criteria assigned according to current CLSI VET01-S veterinary breakpoints.

#### Safety assessment

All dogs receiving at least one administration of a product comprised the safety population. At each visit, the veterinarian performed a general physical examination of the dogs. The owner reported any abnormal observations.

Particular attention was focused on apparent hearing loss. At each visit, a clap test was performed by the veterinarian to estimate the hearing capacity of the dog, in addition to the owner's observations.

Safety parameters were analyzed descriptively without any analysis.

### Statistical analysis

The sample-size calculation was based on the expected percentage change from baseline in the OTIS3 score following treatment. Expected OTIS3 score evolution and variability were derived from previous clinical data for Easotic™ (15). A 15-percentage-point between-treatment difference was used as the target difference for the calculation, assuming a common standard deviation of 25%. Under these assumptions, 45 evaluable dogs per treatment group were required to provide 80% power at a two-sided significance level of 0.05. The protocol aimed to obtain approximately 50 evaluable dogs per treatment group and allowed the randomization of up to 120 dogs to account for loss to follow-up, early withdrawal, protocol deviations, and other causes of non-evaluability. Ultimately, 86 dogs were randomized and 80 constituted the Day-0 PP population; therefore, the planned number of evaluable dogs was not achieved. Because the achieved sample size fell short of the initial power calculation, the resulting statistical estimates and associated confidence intervals, most notably within the PP population, exhibit a wider margin of error. Consequently, these point estimates should be interpreted in light of the reduced statistical power and diminished estimator precision.

The primary OTIS3 efficacy variables specified in the protocol were the post-treatment OTIS3 total score and its percentage change from baseline. Both variables were evaluated using repeated-measures models, with between-treatment comparisons performed overall and at each post-treatment visit in the ITT and PP populations. The protocol did not designate a single combination of endpoint, visit, analysis population, and treatment contrast as the sole primary analysis. Because the study aimed to evaluate the onset of treatment activity, particular attention was given to the planned Day-2 comparison. However, Day 2 was not prespecified as the sole primary time point, and the Day-2 ITT comparison should therefore not be interpreted as a uniquely prespecified confirmatory primary analysis. Additional clinical parameters were considered secondary efficacy endpoints.

All statistical analyses were performed using R software, version 4.2.3 (R Foundation for Statistical Computing, Vienna, Austria). All tests were two-sided, with statistical significance set at α = 0.05. Reported *p*-values were not adjusted for multiple comparisons, and all secondary comparisons were considered exploratory. Continuous demographic and baseline variables were summarized using the number of observations, mean, standard deviation, median, range, and 95% confidence interval (CI), while categorical variables were summarized using frequencies and percentages.

The primary continuous efficacy endpoints, comprising the OTIS3 total score and its percentage change from baseline, were analyzed under the missing-at-random assumption using linear mixed-effects repeated-measures models fitted with the lme function from the nlme package. Treatment group, visit, and the treatment-by-visit interaction were included as fixed effects, with baseline OTIS3 score included as a covariate. Random intercepts were included for study site/veterinarian and dog, with dogs nested within study sites. Repeated observations within dogs were modeled using a first-order autoregressive correlation structure implemented with the corAR1 function. Homogeneous and visit-specific heterogeneous residual variance structures were evaluated, the latter using the varIdent function, and the structure yielding the lowest Akaike information criterion (AIC) was retained.

Model assumptions were assessed by visual inspection of Pearson residuals plotted against fitted values and normal Q–Q plots. Residual distributions, variance patterns, and potentially influential observations were also examined. Adjusted least-squares (LS) means and between-treatment contrasts at each visit were estimated using the emmeans and contrast functions from the emmeans package. Degrees of freedom were calculated using the containment method implemented for nlme models. Treatment effects are reported as adjusted LS-mean differences with corresponding 95% CIs and two-sided *p*-values. No multiplicity adjustment was applied to the visit-specific comparisons.

The same Gaussian repeated-measures framework was applied to changes from baseline in the individual OTIS3 component scores (erythema, oedema, discharge, and erosion) and veterinarian-assessed secondary clinical parameters recorded on four-level scales ranging from 0 to 3. Because of the ordinal nature of these variables, cumulative link mixed models with a logit link were additionally fitted as sensitivity analyses using the clmm function from the ordinal package. Separate ordinal models were fitted for each outcome and included baseline score, treatment group, visit, and the treatment-by-visit interaction as fixed effects, with random intercepts for study site/veterinarian and dog. Adjusted between-treatment contrasts at each visit were estimated using the emmeans and contrast functions and are reported as cumulative odds ratios with 95% CIs and two-sided *p*-values. An odds ratio below 1 for Easotic^TM^ vs. Claro^TM^ indicated lower odds of being classified in a higher, less favorable score category with Easotic^TM^. Model convergence was assessed using the optimizer convergence information and maximum absolute gradient. These ordinal sensitivity analyses were performed in both the ITT and PP populations, and their conclusions were compared with those obtained from the Gaussian models.

Changes in cytological scores at Days 7 and 28 were analyzed separately using linear mixed-effects models fitted with the lmer function from the lme4 package, with treatment as a fixed effect and study site/veterinarian as a random effect. Binary outcomes, including treatment success at Days 7 and 28, clinical success, and relapse rate, were analyzed using generalized linear mixed-effects models with a binomial distribution and logit link, fitted with the glmer function from the lme4 package. Treatment was included as a fixed effect and study site/veterinarian as a random effect, and treatment effects are reported as adjusted odds ratios with 95% CIs.

Ordinal global assessments of treatment response by owners and veterinarians were analyzed separately using cumulative link mixed models fitted with the clmm function from the ordinal package, with treatment as a fixed effect and study site/veterinarian as a random effect. Microbiological culture and antimicrobial susceptibility data were summarized descriptively.

All primary efficacy inferences and clinical conclusions are based on the Intention-To-Treat (ITT) population. The Per-Protocol (PP) population was evaluated secondary as an exploratory sensitivity analysis to examine treatment effect consistency under protocol-adhered, label-aligned conditions.

## Results

### Populations

The dogs were enrolled at 7 clinical study sites. Dogs were randomly assigned (1:1) in chronological order of presentation to receive either Easotic^TM^ or Claro^TM^. Randomization was managed electronically using a dynamic, “just-in-time” system without *post-hoc* re-balancing. Treatment assignments were generated at the moment of enrollment, preventing selection bias and maintaining blinding. Following written owner informed consent, 86 dogs meeting all baseline inclusion criteria were randomized across the trial (43 assigned to the Easotic group and 43 to the Claro group), forming the complete *Intention To Treat* (ITT) population.

The post-randomization exclusions for the secondary exploratory sensitivity analyses allowed the establishement of the Per Protocol population. The Per Protocol population included 80 dogs (41 dogs in the Easotic group and 39 dogs in the Claro group).

In the Easotic group, two (2) dogs were withdrawn due to owner non-compliance (absence of treatment administration after Day 0 and failure to attend the scheduled Day 7 visit). In the Claro group, four (4) dogs were excluded following baseline cytological examination, which revealed rod-shaped bacteria exclusively.

This field trial was specifically designed to mirror daily veterinary practice as closely as possible. The dual-population presentation aimed to reflect two common real-world practice scenarios. The ITT population reflects empirical field practice where the veterinarian initiates treatment based on clinical signs and veterinarian expertise without performing prior cytology (e.g., uncooperative dog, lack of owner adherence, or financial constraints). The PP population reflects label-aligned practice where a veterinarian performs in-clinic cytology prior to treatment selection.

Among the 86 included dogs, 36 breeds were represented. The mixed breeds were most common (23%). Labrador Retriever (9%), Golden Retriever (8%), Poodle (8%) and German Shepherd (7%) were the other most common breeds seen in the study. The Easotic group contained 20 left ears and 23 right ears, while the Claro group included 17 left ears and 26 right ears. The average age was 5.5 years in the Claro group and 5 years in the Easotic group. For both groups, the average weight was 27 kg (range: 3–59 kg). Male dogs represented 55% of the dogs enrolled (77% of them neutered) and female dogs 45% (79% of them neutered). Baseline sex distribution differed numerically between groups (Easotic™: 42% male, 58% female; Claro™: 67% male, 33% female). This numerical imbalance is acknowledged as a baseline demographic variance. However, because topical otic formulations exert localized activity directly within the external ear canal without relying on systemic absorption or sex-dependent physiological metabolism, this baseline variation is not considered a biologically plausible confounder of efficacy. Sex was not prespecified as a covariate in the trial protocol and was therefore not included in the primary statistical modeling.

The distribution of enrolled dogs is detailed in the table provided in the Supplementary material 1.

The distribution by study site is detailed in the table provided in the Supplementary material 2.

### Clinical evaluation for intention to treat population (*n* = 86)

#### OTIS3 scores

The mean OTIS3 scores decreased from 7.7 (SD = 1.53; min 5.0; max 11.0) (Easotic group) and 7.6 (SD = 1.42; min 8.0; max 11.0) (Claro group) at Day 0 to respectively, 5.3 (95%: 4.87; 5.77) and 5.2 (95%CI: 4.73; 5.63) (*p* = 0.628) at D0+4h, 3.2 (95%: 2.72; 3.73) and 4.2 (95%: 3.69; 4.72) at Day 2 (*p* = 0.003), 1.8 (95%: 1.25; 2.38) and 2.8 (95%: 2.19; 3.31) at Day 7 (*p* = 0.010) and 1.4 (95%: 0.78; 2.03) and 2.0 (95%: 1.43; 2.69) at Day 28 (*p* = 0.108).

In the adjusted repeated-measures analysis, the between-treatment difference in OTIS3 total score (Easotic^TM^ minus Claro^TM^) was 0.14 points at Day 0+4h (95% CI: −0.43 to 0.71; *p* = 0.628), −0.98 points at Day 2 (95% CI: −1.62 to −0.33; *p* = 0.003), −0.94 points at Day 7 (95% CI: −1.65 to −0.23; *p* = 0.010), and −0.65 points at Day 28 (95% CI: −1.44 to 0.14; *p* = 0.108).

The reduction from baseline in OTIS3 scores was significantly greater in the Easotic-treated dogs than in the Claro-treated dogs at Day 2 (59% and 46% respectively, *p* = 0.004) and at Day 7 (77% and 65% respectively, *p* = 0.015). At Day 2, the adjusted between-treatment difference in percentage reduction was 12.71 percentage points in favor of Easotic^TM^ (95% CI: 4.06 to 21.36; *p* = 0.004).

The mean of OTIS3 score and the percentage of decrease from baseline in both groups is presented in Figure 1.

**Figure 1:**
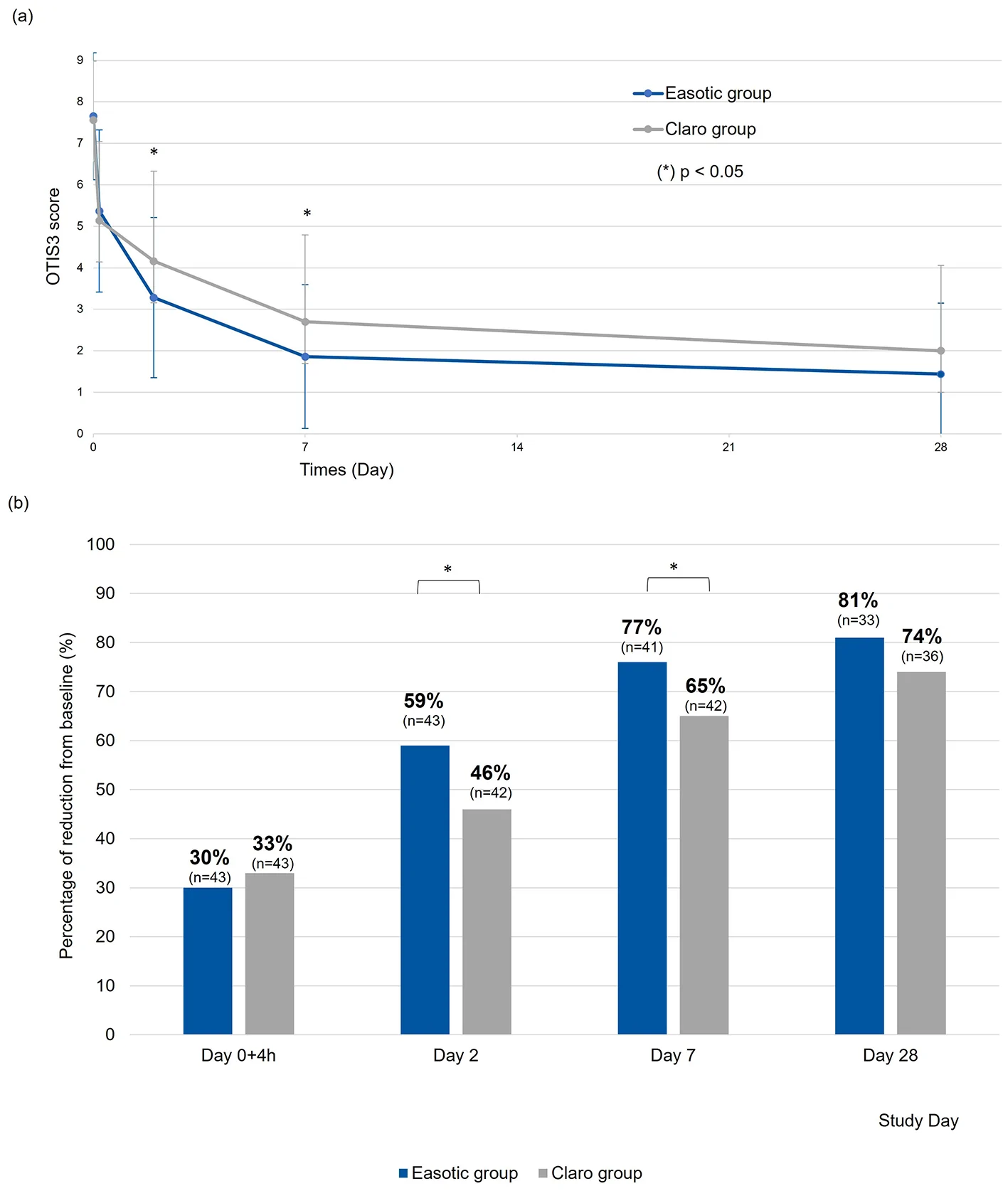
OTIS3 score in each group for ITT population: **(a)** mean of OTIS3 (Error bars = 95% CI) **(b)** percentage of decrease from baseline.

#### Treatment success

On Day 7, treatment success was observed on 85% of dogs in the Easotic group and 74% of dogs in the Claro group (*p* = 0.197). On Day 28, treatment success rose to 85% in the Easotic group and 79% in the Claro group (*p* = 0.400).

#### OTIS3 components

At Day 2 and Day 7, the score decrease from baseline for ear discharge was nominally higher in the Easotic group than the Claro group. On Day 2, the ear discharge decreased by 1.3 (95% CI: −1.49; −1.17) in the Easotic group and by 0.9 (95% CI: −1.06; −0.74) in Claro group compared to Day 0 (*p* < 0.001). At Day 7, the ear discharge decreased by 1.6 (95% CI: −1.80; −1.43) in the Easotic group and by 1.1 (95% CI: −1.29; −0.91) in the Claro group compared to Day 0 (*p* < 0.001). At Day 28, the ear discharge decrease remained higher in the Easotic group (by 1.6; 95% CI: −1.78; −1.38) than in the Claro group (by 1.3; 95% CI: −1.54; −1.14) (*p* = 0.068).

The change in scores from baseline, for erythema, oedema, and erosion were similar between the Easotic group and the Claro group on each post-treatment study day.

#### Secondary veterinarian ear assessment

On Day 2, the stenosis score decrease from baseline was nominally higher in the Easotic group (by 0.7; 95% CI: −0.82; −0.54) than the Claro group (by 0.5; 95% CI: −0.60; −0.32) (*p* = 0.016).

The odor score and the dirtiness score decrease from baseline were nominally higher in the Easotic group than in the Claro group from Day 2.

From Day 0 to Day 2, ear odor decreased by 1.3 (95% CI: −1.46; −1.16) in the Easotic group and by 1.0 (95% CI: −1.17; −0.87) in Claro group (*p* = 0.001) and the ear dirtiness by 1.3 (95% CI: −1.43; −1.10 and by 1.0 (95% CI: −1.21; −0.84), respectively (*p* = 0.002). From Day 0 to Day 7, the ear odor decreased by 1.4 (95% CI: −1.59; −1.22) in the Easotic group and 1.0 (95% CI: −1.23; −0.86) in the Claro group (*p* < 0.001) and the ear dirtiness by 1.5 (95% CI: −1.67; −1.30) and by 1.0 (95% CI: −1.21; −0.83), respectively (*p* < 0.001).

At Day 28, the decrease of ear odor and ear dirtiness remained higher in the Easotic group by 1.4 (95% CI: 1.63; −1.23) and 1.5 (95% CI: −1.71; −1.28), respectively, compared to the Claro group with 1.2 (95% CI: −1.42; −0.99) and 1.2 (95% CI:−1.40; −1.00) respectively (*p* = 0.050 and *p* = 0.028, respectively).

For ear pain, the score decrease from baseline was similar between the Easotic group and the Claro group on each post-treatment study day.

#### Veterinarian overall assessment of response to treatment

At Day 7, veterinarians subjectively assessed the response to treatment as *Excellent* (complete resolution of the initial clinical signs) in a nominally higher percentage of dogs in the Easotic group (50%) as compared to the Claro group (26%) (*p* = 0.009) (Figure 2). At Day 7, veterinarians assessed the response to Easotic^TM^ treatment as *Excellent* or *Good* in 90% of dogs (69% for Claro^TM^, *p* = 0.006).

**Figure 2:**
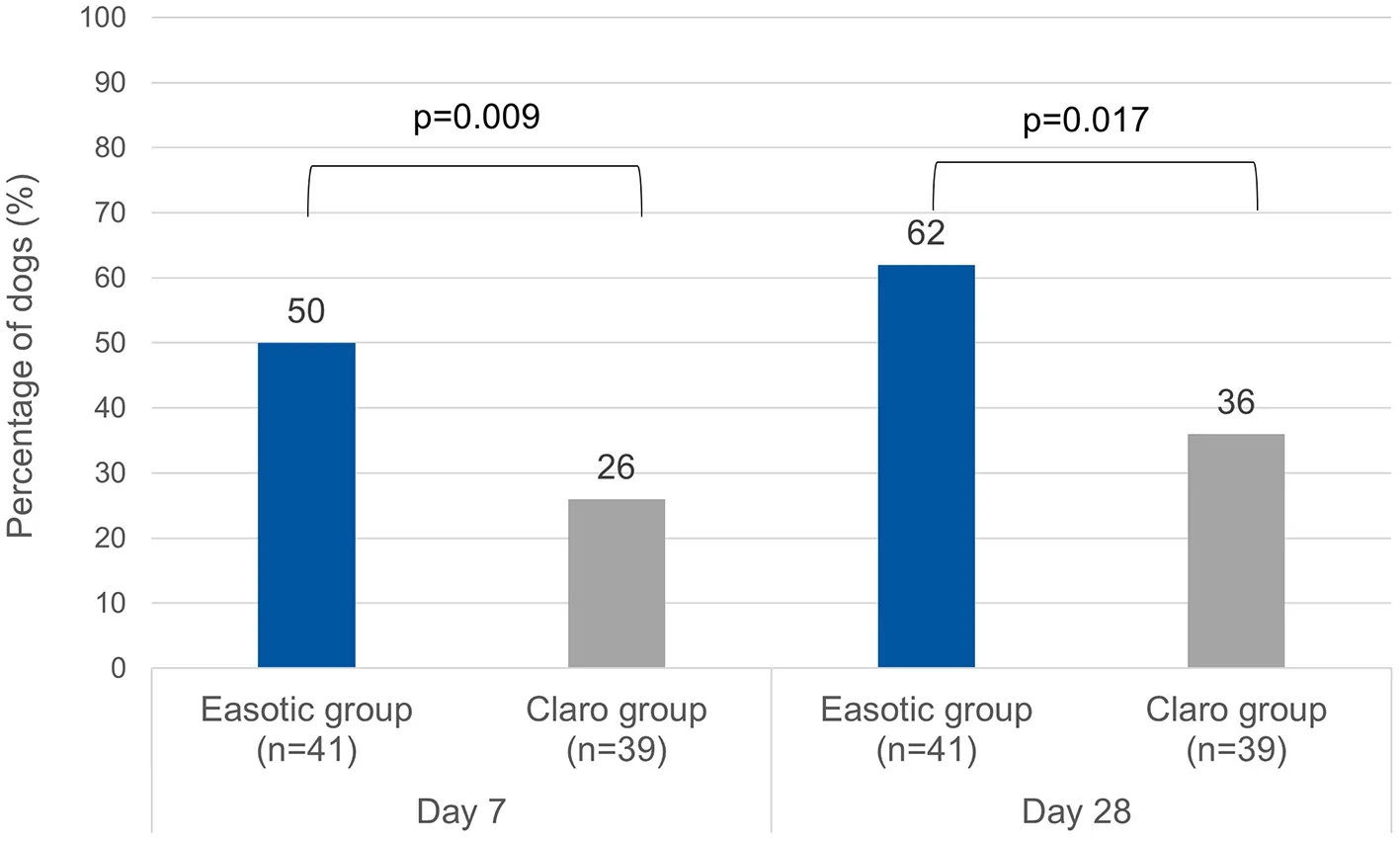
Percentage of dogs showing an Excellent response to treatment according to the veterinarian in each group for ITT population.

At Day 28, veterinarians assessed the response to treatment as *Excellent* for a nominally higher percentage of dogs in the Easotic group (62%) compared to the Claro group (36%) (*p* = 0.017) (Figure 2). On Day 28, veterinarians assessed the response to Easotic^TM^ treatment as *Excellent* or *Good* for 86% of dogs (76% for Claro^TM^; *p* = 0.020).

#### Owner assessment

According to pet owners, from Day 2, the decrease of ear odor and ear dirtiness remained higher after Easotic^TM^ treatment than Claro treatment. At Day 7, the ear odor was decreased after administration of Easotic^TM^ compared to Claro^TM^ (*p* = 0.034).

From Day 7, the decrease of ear pain and ear pruritus remained higher after Easotic^TM^ treatment than Claro treatment. At Day 7, the ear pain was decreased after administration of Easotic^TM^ compared to treatment with Claro^TM^ (*p* = 0.038).

At Day 7, the improvement of the otitis manifestations after Easotic^TM^ administration was considered as *Excellent or Good* according to 91 % of the owners compared to 88% in the Claro group (*p* = 0.205).

The product characteristics were evaluated as *Excellent* or *Good* for a similar proportion of owners in both groups (96% for Easotic^TM^ and 91% for Claro^TM^; *p* = 0.179).

#### Cytological score

At Day 0, the mean cytological score was 4.93 (SD = 1.80; min 1.00; max 8.00) in the Easotic group and 4.98 (SD = 1.60; min 3.00; max 11.00) in the Claro group. In the Easotic group, cocci were identified by the veterinarian in 93% of dogs, rods in 21% and *yeast* in 72%. In the Claro group, cocci were identified by the veterinarian in 84% of dogs, rods in 30% and *yeast* in 77%.

In the Easotic group, the mean bacteria score was 1.95 (SD = 0.92; min 0.00; max 4.00), the mean yeast score 2.27 (SD = 1.27; min 0.00; max 4.00) and the mean inflammatory cells 0.81 (SD = 1.07; min 0.00; max 4.00).

In the Claro group, the mean bacteria score was 2.21 (SD = 1.21; min 0.00; max 4.00), the mean yeast score was 2.05 (SD = 1.34; min 0.00; max 4.00) and the mean inflammatory cells 0.72 (SD = 0.98; min 0.00; max 4.00).

At Day 7, the decrease of the cytological score from Day 0 was greater in the Easotic group (−71.6%; 95% CI: −0.803; −0.628) than in the Claro group (−57.2%; 95% CI: −0.659; −0.484) (*p* = 0.023).

In the Easotic group, the mean cytological score was 1.35 (SD = 1.31; min 0.00; max 6.00). The mean bacteria, yeast and inflammatory cells scores were respectively 0.56 (SD = 0.70; min 0.00; max 2.00), 0.67 (SD = 0.84; min 0.00; max 4.00), 0.12 (SD = 0.45; min 0.00 max 2.00).

In the Claro group, the mean cytological score was 2.19 (SD = 1.92; min 0.00; max 8.00). The bacteria, yeast, inflammatory cells scores were, respectively 0.88 (SD = 1.10; min 0.00; max 4.00), 1.00 (SD = 0.95; min 0.00, max 3.00), 0.30 (SD = 0.83; min 0.00; max 4.00).

At Day 28, the decrease of the cytological score from Day 0 was similar in both groups and for both populations (−64.4% (95% CI: −0.774; −0.515) and −64.9% (95% CI: −0.779; −0.519), respectively).

In the Easotic group, the mean cytological score was 1.44 (SD = 1.31; min 0.00; max 6.00). The mean bacteria, yeast and inflammatory cells scores were, respectively 0.47 (SD = 0.58; min 0.00; max 2.00), 0.77 (SD = 0.95; min 0.00; max 4.00), 0.14 (SD = 0.47; min 0.00 max 2.00).

In the Claro group, the mean cytological score was 1.77 (SD = 1.81; min 0.00; max 8.00). The bacteria, yeast, inflammatory cells scores were, respectively 0.63 (SD = 1.05; min 0.00; max 3.00), 0.84 (SD = 1.00; min 0.00, max 3.00), 0.26 (SD = 0.82; min 0.00; max 4.00).

### Exploratory sensitivity analysis for the per protocol population (*n* = 80)

#### OTIS3 scores

The mean OTIS3 scores decreased from 7.7 (SD = 1.56; min 5.0; max 11.0) in Easotic group and 7.4 (SD = 1.33; min 5.0; max 11.0) in Claro group at Day 0, respectively to 5.4 (95% CI: 4.95; 5.84) and 4.9 (95% CI: 4.49; 5.40) (*p* = 0.134) at Day 0+4h, 3.3 (95% CI: 2.76; 3.78) and 3.9 (95% CI: 3.39; 4.43) at Day 2 (*p* = 0.064), 1.8 (95% CI: 1.22; 2.34) and 2.5 (95% CI: 1.93; 3.09) at Day 7 (*p* = 0.056), and 0.9 (95% CI: 0.26; 1.47) and 1.4 (95% CI: 0.84; 2.03) at Day 28 (*p* = 0.153).

In the adjusted repeated-measures analysis, the between-treatment difference in OTIS3 total score (Easotic^TM^ minus Claro^TM^) was 0.45 points at Day 0+4h (95% CI: −0.14 to 1.04; *p* = 0.134), −0.64 points at Day 2 (95% CI: −1.31 to 0.04; *p* = 0.064), −0.73 points at Day 7 (95% CI: −1.48 to 0.02; *p* = 0.056), and −0.57 points at Day 28 (95% CI: −1.36 to 0.21; *p* = 0.153).

The reduction percentages from baseline in OTIS3 scores were greater in the Easotic group than in the Claro group from Day 2. At Day 2, the Easotic-treated dogs exhibited a 58% decrease compared to a 50% decrease in the Claro group (*p* = 0.070). At Day 2, the adjusted between-treatment difference in percentage reduction was 8.48 percentage points in favor of Easotic^TM^ (95% CI: −0.70 to 17.66; *p* = 0.070).

The mean of OTIS3 scores and the percentage of decrease from baseline in both groups are presented in Figure 3.

**Figure 3:**
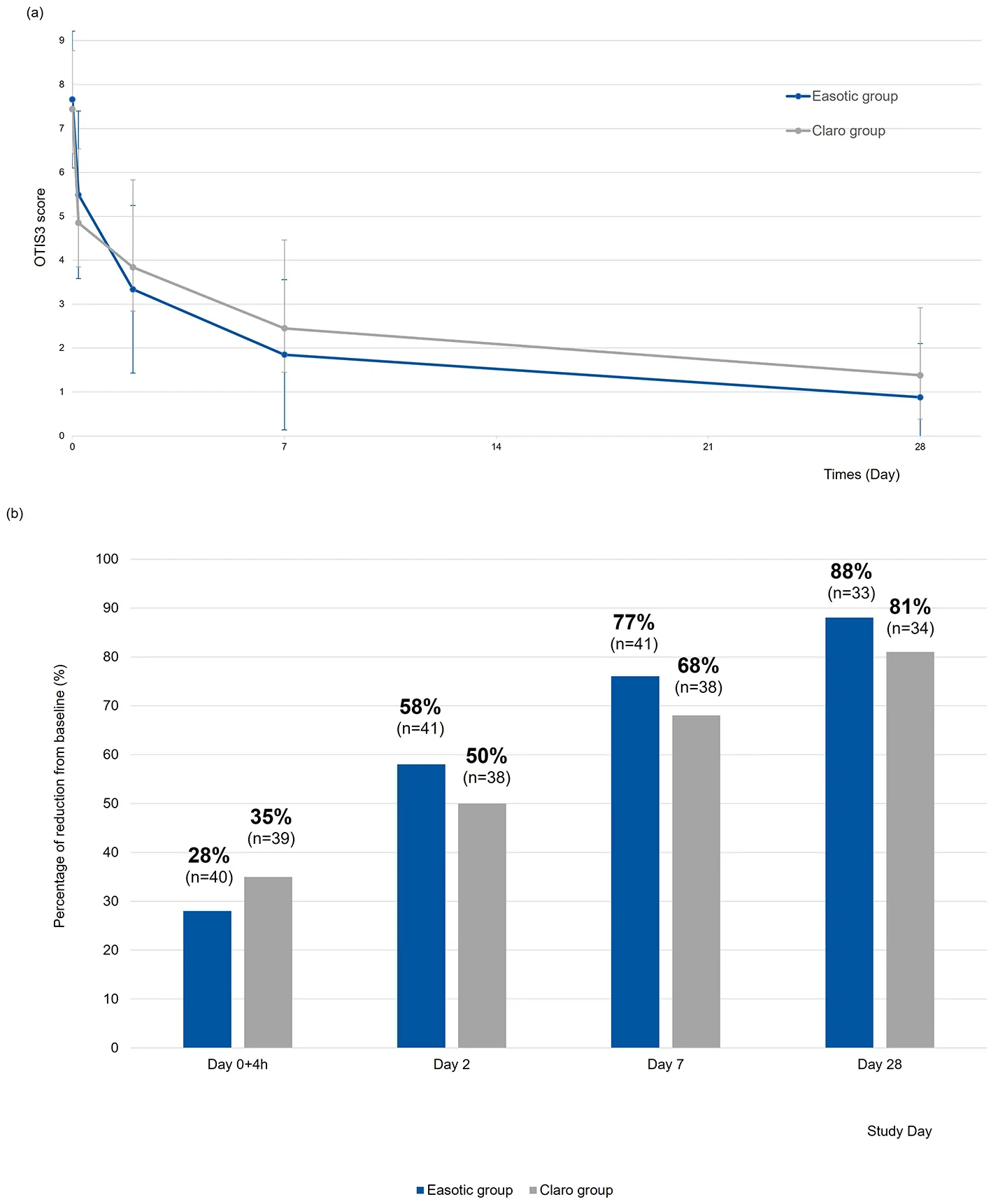
OTIS3 score in each group for per protocol population (exploratory sensitivity analyses): **(a)** mean of OTIS3 (Error bars = 95% CI) **(b)** percentage of reduction from baseline.

#### Treatment success

On Day 7, treatment success was observed in 85% of dogs in the Easotic group and in 79% of dogs in the Claro group (*p* = 0.488). On Day 28, treatment success rose to 94% in the Easotic group and 88% in the Claro group (*p* = 0.261) (Figure 4).

**Figure 4:**
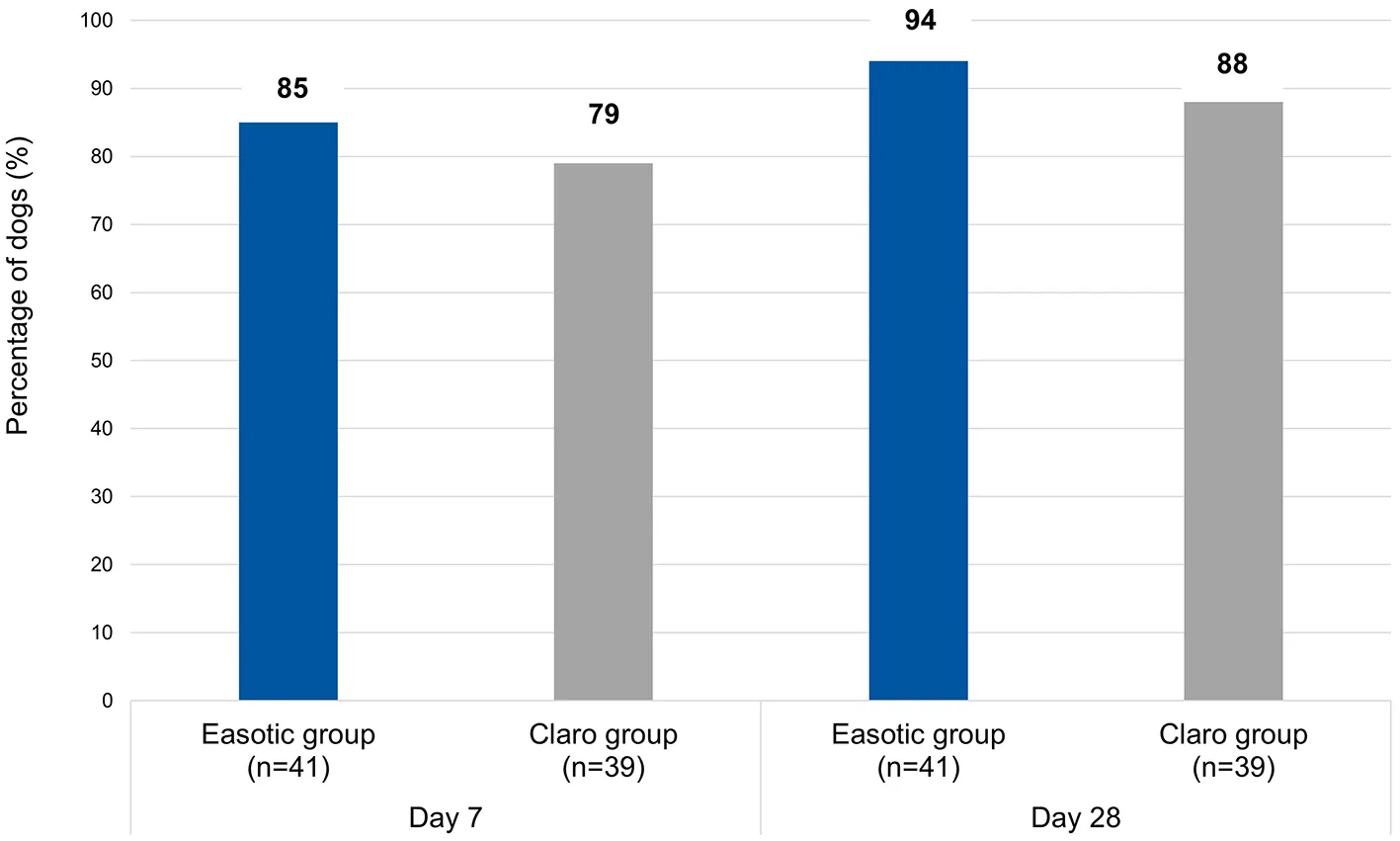
Percentage of dogs showing a reduction of the OTIS3 score from ≥5 at Day 0 to ≤ 3 at Day 7 and Day 28 (treatment success) for per protocol population (exploratory sensitivity analyses).

#### OTIS3 components

At Day 2 and Day 7, the decrease of scores from baseline for ear discharge was nominally higher in the Easotic group compared to the Claro group. At Day 2, the ear discharge decreased by 1.3 (95% CI: −1.51; −1.11) in the Easotic group and by 0.9 (95% CI: −1.15; −0.74) in the Claro group compared to Day 0 (*p* = 0.005). At Day 7, the ear discharge decreased by 1.6 (95% CI: −1.81; −1.36) in the Easotic group and by 1.1 (95% CI: −1.33; −0.85) in the Claro group compared to Day 0 (*p* = 0.001). At Day 28, the ear discharge decrease remained higher in the Easotic group (by 1.8) compared to the Claro group (by 1.5) (*p* = 0.069).

The change of scores from baseline, for erythema, oedema, and erosion were similar between the Easotic group and Claro group on each post-treatment study day.

The score decrease from baseline is presented in Figure 5.

**Figure 5:**
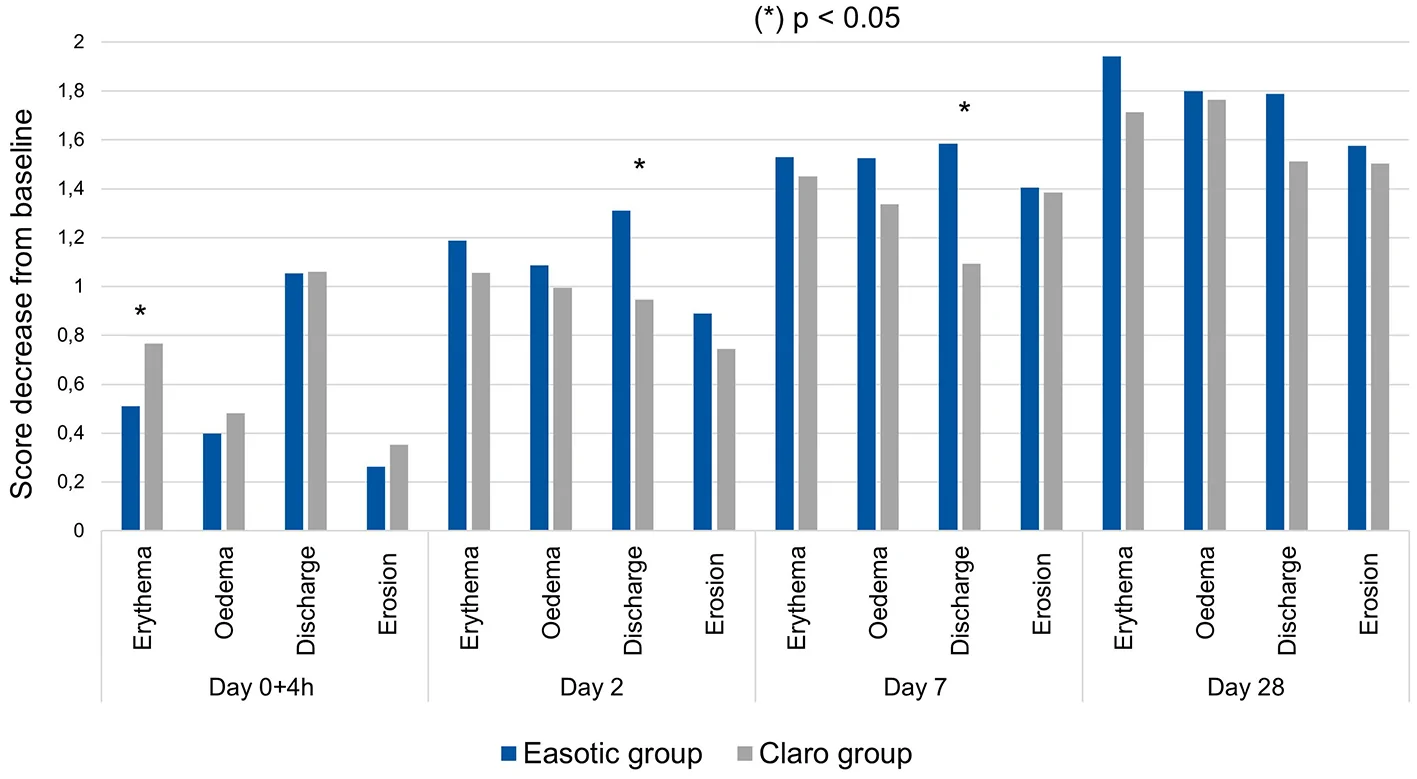
OTIS3 components: score decrease from baseline in each group for per protocol population (exploratory sensitivity analyses).

#### Secondary veterinarian ear assessment

At Day 2, the decrease in stenosis score was nominally greater in the Easotic group compared to the Claro group. From Day 0 to Day 2, the ear stenosis decreased by 0.7 (95% CI: −0.82; −0.53) in the Easotic group and by 0.5 (95% CI: −0.62; −0.33) in the Claro group (*p* = 0.039).

For odor and dirtiness, the decrease from baseline was nominally higher in the Easotic group than in the Claro group at Day 2 and Day 7. From Day 0 to Day 2, the ear odor decreased by 1.3 (95% CI: −1.45; −1.12) in the Easotic group and by 1.0 (95% CI: −1.19; −0.86) in the Claro group (*p* = 0.008) and the ear dirtiness by 1.2 (95% CI: −1.40; −1.06) and by 1.0 (95% CI: −1.18; −0.83), respectively (*p* = 0.030). From Day 0 to Day 7, the ear odor decreased by 1.4 (95% CI: −1.52; −1.19) in the Easotic group and 1.1 (95% CI: −1.27; −0.93) in the Claro group (*p* = 0.011) and the ear dirtiness by 1.5 (95% CI: −1.67; −1.28) and by 1.0 (95% CI: −1.26; −0.86), respectively (*p* < 0.001).

For ear pain, the score decrease from baseline was similar between the Easotic group and the Claro group on each post-treatment study day.

The score decrease from baseline for secondary clinical ear signs assessment by veterinarians is represented in Figure 6.

**Figure 6:**
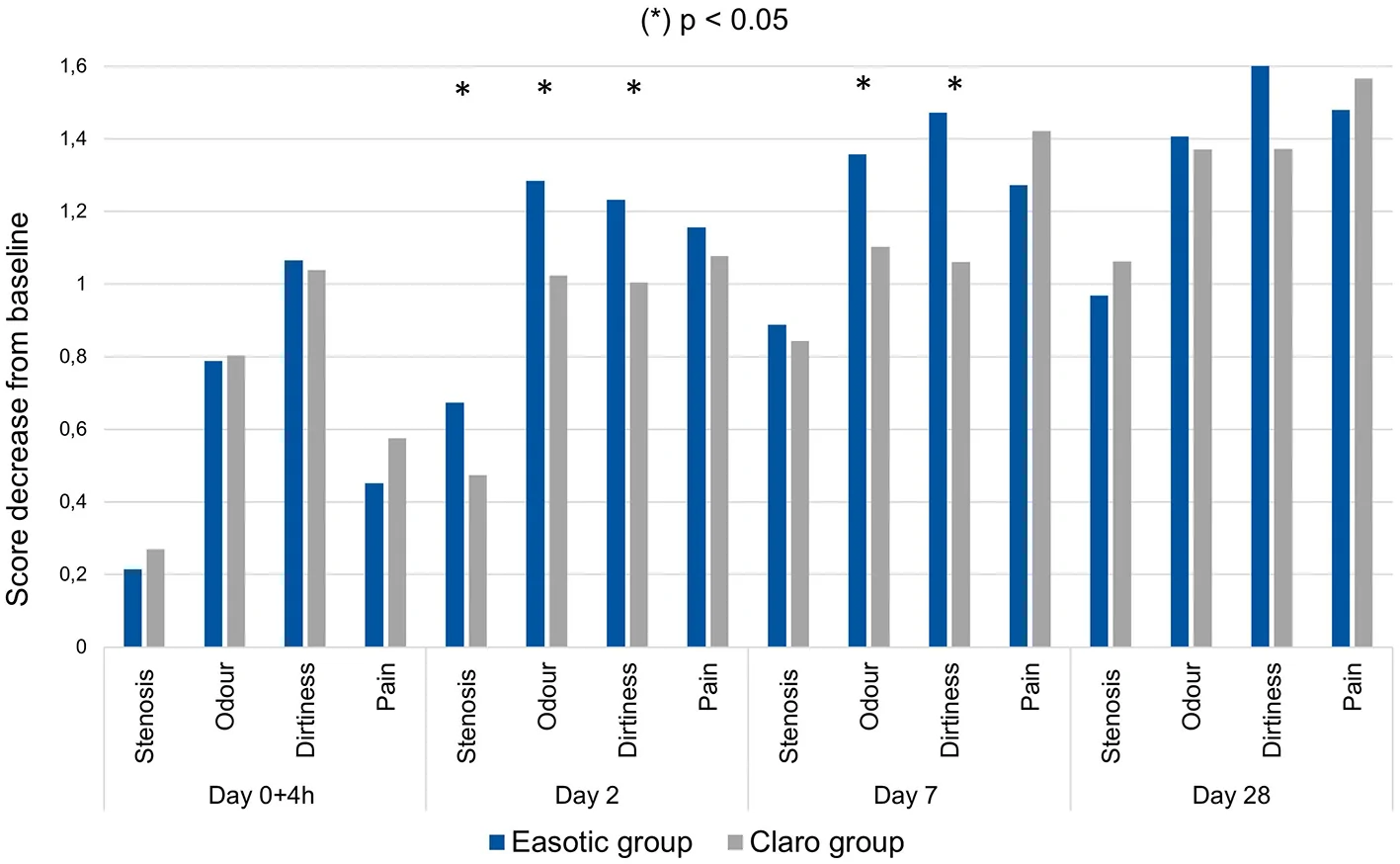
Secondary ear signs: score decrease from baseline in each group for per protocol population (exploratory sensitivity analyses).

#### Veterinarian overall assessment of response to treatment

At Day 7, veterinarians subjectively assessed the response to treatment as *Excellent* (complete resolution of the initial clinical signs) in a nominally higher percentage of dogs in the Easotic group (49%) as compared to the Claro group (29%) (*p* = 0.010) (Figure 7). At Day 7, veterinarians assessed the response to Easotic^TM^ treatment as *Excellent* or *Good* for 90% of dogs (71% for Claro^TM^).

**Figure 7:**
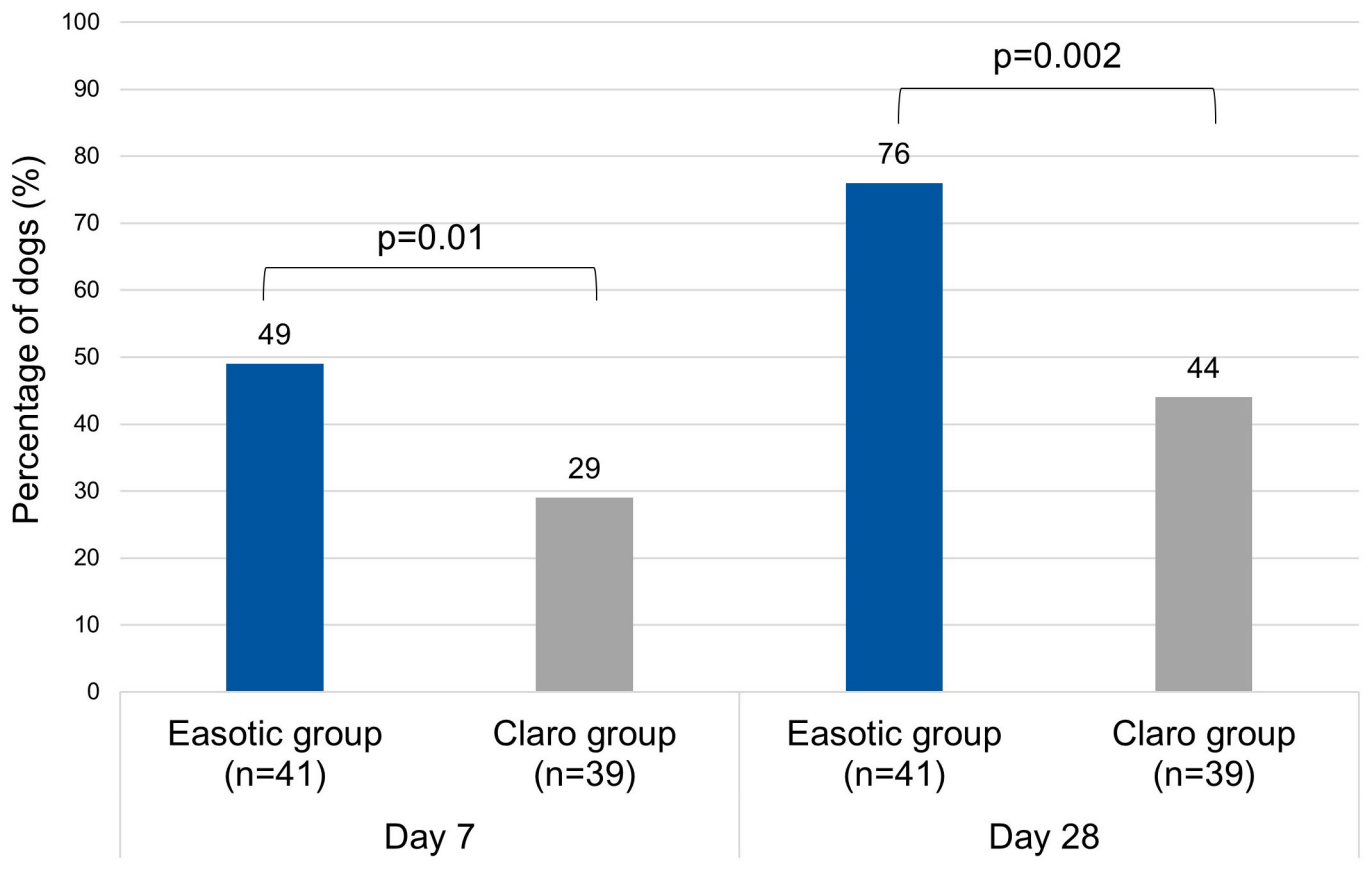
Percentage of dogs showing a complete resolution of the initial clinical signs according to the subjective assessment of the veterinarian in each group for the per protocol population (exploratory sensitivity analyses).

At Day 28, veterinarians assessed the response to treatment as *Excellent* for a nominally higher percentage of dogs in the Easotic group (76%) compared to the Claro group (44%) (*p* = 0.002). At Day 28, veterinarians assessed the response to Easotic^TM^ treatment as *Excellent* or *Good* for 94% of dogs (85% for Claro^TM^).

#### Owner assessment

According to pet owners, from Day 2, the decrease of ear odor, ear pruritus and ear dirtiness remained higher after Easotic^TM^ treatment than treatment with Claro^TM^ (Figure 8).

**Figure 8:**
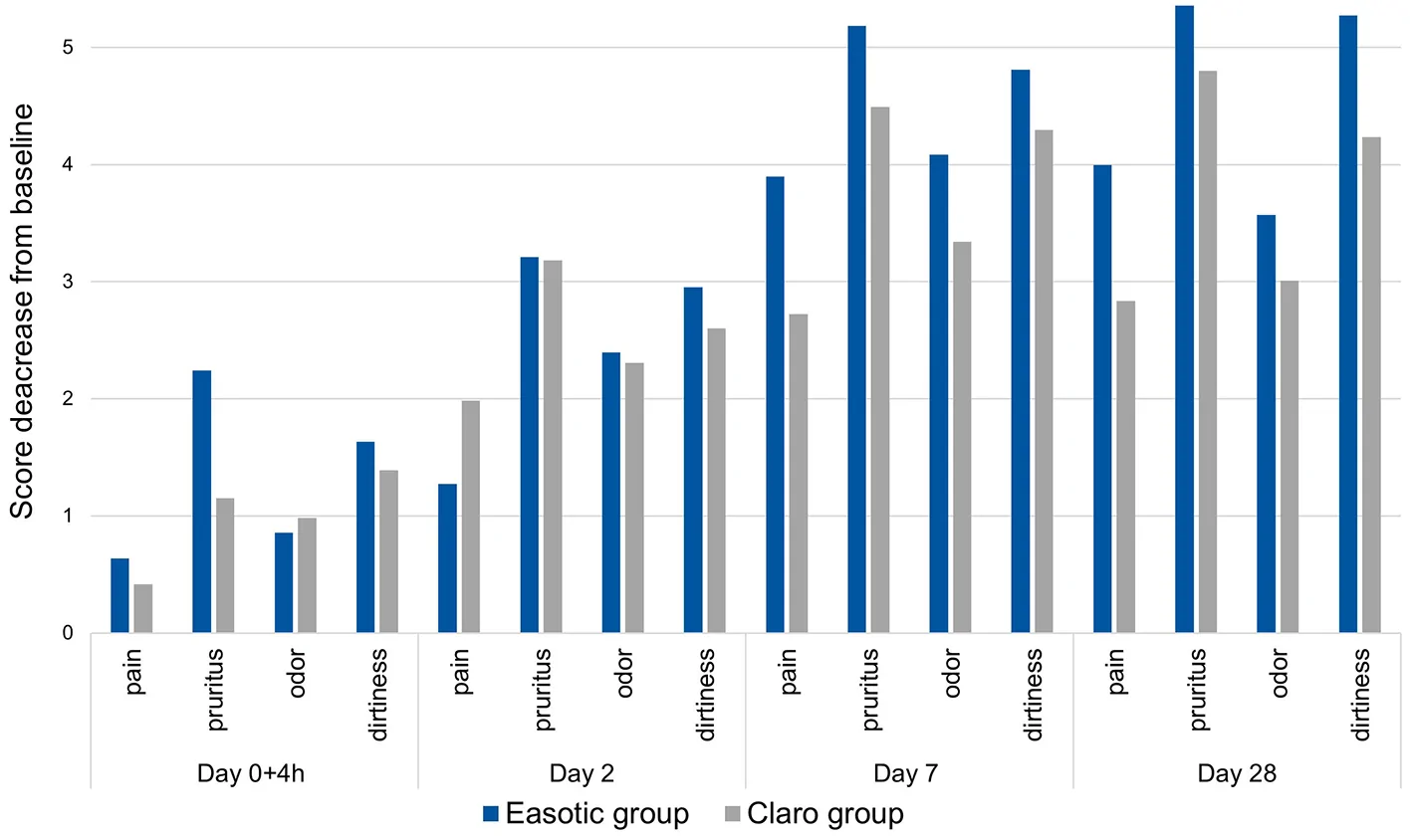
Ear signs owners' assessment: score decrease from baseline in each group for per protocol population (exploratory sensitivity analyses).

At Day 7, the improvement of the otitis manifestations after Easotic^TM^ administration was considered *Excellent* or *Good* according to 91 % of the owners compared to 89% in the Claro group (*p* = 0.266).

The product characteristics were evaluated as *Excellent* or *Good* for a similar proportion of owners in both treatment groups [96% for Easotic^TM^ and 95% for Claro^TM^ (*p* = 0.310)].

#### Cytological score

At Day 0, the mean cytological score was 4.88 (SD = 1.83; min 1.00; max 8.00) in the Easotic group and 4.90 (SD = 1.55; min 3.00; max 11.00) in the Claro group. In the Easotic group, cocci were identified by the veterinarian in 93% of dogs, rods in 22%, and *yeast* in 71%. In the Claro group, cocci were identified by the veterinarian in 92% of dogs, rods in 23%, and *yeast* in 85%.

In the Easotic group, the mean bacteria score was 1.85 (SD = 0.94; min 0.00; max 4.00), the mean yeast score 2.20 (SD = 1.27; min 0.00; max 4.00) and the mean inflammatory cells 0.83 (SD = 1.09; min 0.00; max 4.00).

In the Claro group, the mean bacteria score was 2.03 (SD = 1.11; min 0.00; max 4.00), the mean yeast score was 2.26 (SD = 1.23; min 0.00; max 4.00) and the mean inflammatory cells 0.62 (SD = 0.78; min 0.00; max 3.00).

At Day 7, the decrease of the cytological score from Day 0 was greater in the Easotic group (−72.7%; 95% CI: −0.808; −0.646) than in the Claro group (−61%; 95%CI: −0.699; −0.537) (p = 0.061).

In the Easotic group, the mean cytological score was 1.27 (SD = 1.10; min 0.00; max 4.00). The mean bacteria, yeast and inflammatory cells scores were respectively 0.54 (SD = 0.67; min 0.00; max 2.00), 0.61 (SD = 0.67; min 0.00; max 2.00), 0.12 (SD = 0.46; min 0.00 max 2.00).

In the Claro group, the mean cytological score was 1.89 (SD = 1.52; min 0.00; max 6.00). The bacteria, yeast, inflammatory cells scores were, respectively 0.68 (SD = 0.81; min 0.00; max 3.00), 1.08 (SD = 0.94; min 0.00, max 3.00), 0.13 (SD = 0.41; min 0.00; max 2.00).

At Day 28, the decrease of the cytological score from Day 0 was similar in both groups and for both populations (−70%; 95%: −0.866; −0.539 and −71%; 95% CI: −0.874; −0.547 respectively).

In the Easotic group, the mean cytological score was 1.12 (SD = 0.99; min 0.00; max 3.00). The mean bacteria, yeast and inflammatory cells scores were respectively 0.42 (SD = 0.56; min 0.00; max 2.00), 0.64 (SD = 0.82; min 0.00; max 3.00), 0.06 (SD = 0.24; min 0.00; max 1.00).

In the Claro group, the mean cytological score was 1.38 (SD = 1.16; min 0.00; max 4.00). The mean bacteria, yeast, inflammatory cells scores were, respectively 0.38 (SD = 0.65; min 0.00; max 3.00), 0.97 (SD = 1.03; min 0.00; max 3.00), 0.03 (SD = 0.17; min 0.00; max 1.00).

### Exploratory analysis of baseline microbial susceptibility

Ear swabs sampled at Day 0 were sent to a laboratory for culture and susceptibility testing. The results of the laboratory testing were received after the masked administration of the product. The baseline microbial susceptibility findings were descriptive and exploratory. The proportion of dogs showing laboratory results with an organism resistant to the administered antibiotic component was similar in both groups (12% in the Easotic group and 14% in the Claro group). In the Easotic group, 60% of dogs (3/5) showing bacteria resistant to gentamicin in the baseline laboratory analysis achieved treatment success at Day 7. In the Claro group, 17% of dogs (1/6) showing bacteria resistant to florfenicol in the baseline laboratory analysis achieved treatment success at Day 7.

### Safety evaluation

Both products were well-tolerated. The safety population is identical to the ITT population and comprised 86 dogs (43 dogs in the Easotic group and 43 dogs in the Claro group). In the Easotic group, suspected adverse reactions were observed in four (4) dogs (9.3%). These events included gastrointestinal disorders (*n* = 2), atopic dermatitis with pruritus-associated agitation (*n* = 1), and mild, transient peri-auricular pruritus occurring one day after the initial administration (*n* = 1). In the Claro group, suspected adverse reactions were recorded in two (2) dogs (4.7%), consisting of dermatitis in the axillary region (*n* = 1) and atopic dermatitis (*n* = 1). Auditory function was monitored following product administration using a clap test. In the Easotic group, suspected hearing deficit was noted in two dogs. One dog exhibited pre-existing deafness at baseline (Day 0) that persisted throughout the study. A second dog experienced transient hearing loss 4 hours post-administration (Day0+4 h), which resolved by Day 2. This transient loss coincided with the presence of yellow mucoid exudate in both ear canals at the 4-h time point; the discharge was resolved by Day 2. No hearing loss was reported in the Claro group.

## Discussion

Canine otitis externa represents a prevalent inflammatory disorder of the external ear canal that necessitates targeted, evidence-based management strategies (1, 3, 6, 10). This multi-center, masked clinical field trial assessed the efficacy of two commercial otic products in dogs suffering from otitis externa: a gentamicin, miconazole, and hydrocortisone aceponate otic suspension (Easotic™, Virbac) administered once daily for five days, and a single-dose florfenicol, terbinafine, and mometasone furoate otic solution (Claro™, Elanco).

The dogs were followed for 28 days with an evaluation at Day 0+4 h, Day 2, Day 7 and Day 28. The veterinarians assessed ear status based on the OTIS3 clinical score (ear signs of erythema, oedema/hyperplasia, discharge and erosion/ulcers) alongside additional clinical characteristics (pain, odor, dirtiness and stenosis).

The safety analysis showed that both products were well-tolerated with an absence of serious adverse events. Specifically, no persistent clinically apparent and treatment-related hearing deficit was detected by the screening method used.

The percentage of dogs with a reduction in the OTIS3 score from ≥ 5 at Day 0 to ≤ 3 at Day 7 was higher in the Easotic^TM^ group (85%) than in the Claro^TM^ group (79%) (*p* = 0.488). A reduction of the OTIS3 to a score ≤ 3 at Day 7 was considered a treatment success. Both products were effective in improving the ear status and in OTIS3 score reduction. Furthermore, the reduction of the OTIS3 score was higher for the Easotic^TM^ group compared to the Claro^TM^ group at Day 2, Day 7 and Day 28. The clinical outcomes were aligned with the overall assessment of treatment response by both veterinarians and dog owners. The veterinarians subjectively assessed the response to treatment as “*Excellent*” for a higher number of dogs in the Easotic^TM^ group (49%) than in the Claro^TM^ group (29%) from Day 7 (*p* = 0.010). The dog owners shared a similar opinion with a higher percentage of dog owners in the Easotic^TM^ group characterizing the improvement of ear manifestations as “*Excellent*” or “*Good*” than in the Claro^TM^ group.

While the study provides valuable insights, several limitations warrant consideration. The blinded design limited the investigator biases, however, the color and consistency of the product applied may have been noted at Day 2 and potentially at Day 7. The different mode of administration between the two products, specifically that owners in the Claro^TM^ group were not involved in administering treatment, may have introduced assessment bias in the Easotic^TM^ group via a caregiver placebo effect (16). While active owner involvement can heighten perceived clinical improvement, repeated home administration may also diminish overall convenience compared to a single, clinic-applied dose. Owner-led at-home treatment and monitoring offers valuable potential for tracking clinical progress. Cosmetic outcomes and owner-perceived otitis manifestations, including reduction of dirtiness and odor, are important to dog owners.

The exclusion of dogs according to the cytology results can be seen as a limitation regarding the objective to mirror field conditions. This potential bias was limited by the presentation of the results obtained in the ITT population. In the ITT population, we reported outcomes for all dogs regardless of ear cytological findings. The ITT population results reflect real-world clinical conditions, in which financial or logistical constraints may preclude the routine performance of cytological examinations and bacterial cultures. In this study, baseline otic cytology was central to establishing the secondary Per Protocol population; consequently, these outcomes are interpreted as exploratory sensitivity analyses to support the primary ITT findings. The empirical treatment is typical for otitis externa in practice particularly when occurring acutely (1). Microbiological susceptibility testing may offer limited usefulness in treating otitis externa. In fact, topical therapy is often successful despite resistance seen on microbiological analysis (17, 18). In-clinic cytology provides immediate identification of organisms to guide initial treatment decisions, whereas culture and susceptibility results are unavailable at baseline. In the absence of cytological confirmation, empirical treatment may present a risk of reduced clinical response; therefore, the specific antibiotic class should be considered to support rational product selection. Nevertheless, performing pre-treatment diagnostic cytology remains the gold standard to enable targeted, narrow-spectrum antimicrobial therapy (1, 11).

This study confirmed that both products, Easotic^TM^ and Claro^TM^, were associated with substantial clinical otitis improvement, with no significant difference in Day-28 treatment success.

Easotic^TM^ produced greater early improvements at Day 2, especially with relation to ear discharge, ear stenosis, ear odor and ear dirtiness.

This study confirmed the clinical efficacy of Easotic™ in the management of canine otitis externa, aligning with findings from previous clinical trials (15, 19). Given that inflammation represents a central pathophysiological process of otitis externa and existing literature indicates that hydrocortisone aceponate (HCA) monotherapy achieves efficacy comparable to multi-component formulations in select clinical contexts (20–23), the anti-inflammatory activity of HCA could likely contribute to the overall clinical response. However, because this trial evaluated complete treatment regimens, the specific contribution of individual active ingredients cannot be isolated.

## Conclusions

For canine otitis externa, Easotic™ and Claro™ were associated with substantial clinical improvement. Furthermore, Easotic™ was associated with early improvement in selected ear signs, particularly discharge, stenosis, odor, and dirtiness.

## Data Availability

The raw data supporting the conclusions of this article will be made available by the authors, without undue reservation.

## References

[1] Nuttall T. Managing recurrent otitis externa in dogs: what have we learned and what can we do better? JAVMA. (2023) 261:S10–22. doi: 10.2460/javma.23.01.0002 37019436

[2] Paterson S Matyskiewicz W. A study to evaluate the primary causes associated with pseudomonas otitis in 60 dogs. J Small Anim Pract. (2018) 59:238. doi: 10.1111/jsap.12813 29322531

[3] Bajwa J. Canine otitis externa - treatment and complications. Can Vet J. (2019) 60:97–9. PMC6294027 30651659

[4] Carlotti DN Taillieu-Leroy S. Otitis externa in the dog - etiology and clinical findings, 452 literature review and retrospective study of 752 cases. Prat Med Chir Anim Comp. (1997) 32:243–57.

[5] Secker B Shaw S Atterbury RJ. *Pseudomonas* spp. in canine otitis externa. Microorganisms. (2023) 11:2650. doi: 10.3390/microorganisms11112650 38004662 PMC10673570

[6] Aymeric-Cuingnart E Bensignor E. A prospective study of canine otitis in general practice. Rev et Clin. (2018) 53:3–9. doi: 10.1016/j.anicom.2017.11.001

[7] Bensignor E Legeay D Médaille C. Prospective study on otitis externa in the adult 455 dog in France. Prat Med Chir Anim Comp. (2000) 35:405–14.

[8] Saridomichelakis MN Farmaki R Leontides LS Koutinas AF. Aetiology of canine otitis externa: a retrospective study of 100 cases. Vet Dermatol. (2007) 18:341–7, 460. doi: 10.1111/j.1365-3164.2007.00619.x 17845622

[9] Brazzini B Pimpinelli N. New and established topical corticosteroids in dermatology. Am J Clin Dermatol. (2002) 3:47–58. doi: 10.2165/00128071-200203010-00005 11817968

[10] Di Traglia R Tudor-Green B Muzaffar J Borsetto D Smith ME. Antibiotics versus non-antibiotic treatments for acute otitis externa: a systematic review and meta-analysis. Clin Otolaryngol. (2023) 48:841–62. doi: 10.1111/coa.14084 37550850

[11] Tam VH Kabbara S Vo G Schilling AN Coyle EA. (2006). Comparative pharmacodynamics of gentamicin against *Staphylococcus aureus* and *Pseudomonas aeruginosa*. Antimicrob Agents Chemother. (2006) 50:2626–31. doi: 10.1128/AAC.01165-05 16870751 PMC1538660

[12] Nuttall T Bensignor E. A pilot study to develop an objective clinical score for canine otitis externa. Vet Dermatol. (2014) 25:530–7, e91–2. doi: 10.1111/vde.12163 25130194

[13] Rybníček J Lau-Gillard PJ Harvey R Hill PB. Further validation of a pruritus severity scale for use in dogs. Vet Dermatol. (2009) 20:115–22. doi: 10.1111/j.1365-3164.2008.00728.x 19171021

[14] Budach SC Mueller RS. Reproducibility of a semiquantitative method to assess cutaneous cytology. Vet Dermatol. (2012) 23:426. doi: 10.1111/j.1365-3164.2012.01075.x 22809453

[15] King SB Doucette KP Seewald W Forster SL. A randomized, controlled, single-blinded, multicenter evaluation of the efficacy and safety of a once weekly two dose otic gel containing florfenicol, terbinafine and betamethasone administered for the treatment of canine otitis externa. BMC Vet Res. (2018) 14:307. doi: 10.1186/s12917-018-1627-5 30305092 PMC6180657

[16] vf Conzemius MG Evans RB. Caregiver placebo effect for dogs with lameness from osteoarthritis. J Am Vet Med Assoc. (2012) 241:1314–9. doi: 10.2460/javma.241.10.1314 23113523

[17] Dyar OJ Huttner B Schouten J Pulcini C. What is antimicrobial stewardship? Clin Microbiol Infect. (2017) 23:793–8. doi: 10.1016/j.cmi.2017.08.026 28882725

[18] Loeffler A Cain CL Ferrer L Nishifuji K Varjonene K Papich MG . Antimicrobial use guidelines for canine pyoderma by the international society for companion animal infectious diseases (ISCAID). Vet Dermatol. (2025) 36:234–82. doi: 10.1111/vde.13342 40338805 PMC12058580

[19] Rigaut D Sanquer A Maynard L Reme C. Efficacy of a topical ear formulation with a pump delivery system for the treatment of infectious otitis externa in dogs: a randomized controlled trial. Intern J App Res Vet Med. (2011) 9:15–28. Available online at: https://jarvm.com/articles/Vol9Iss1/index.htm; https://jarvm.com/articles/Vol9Iss1/Vol9%20Iss1Reme.pdf (Accessed June 5, 2026).

[20] Kuwahara T Poincelot L Jasmin P Bidaud A Kinoshita G. Treatment of canine otitis externa: a randomized, double-blinded, clinical field study comparing the hydrocortisone aceponate monotherapy to a multi-agent reference product. Front Vet Sci. (2026) 13:1861746. doi: 10.3389/fvets.2026.1861746 42582328 PMC13457320

[21] Lorente-Mendez C Alonso-Miguel D. Hydrocortisone aceponate for chronic otitis long-term efficacy. Vet Dermatol. (2025) 36:870–78. doi: 10.1111/vde.70008 40757500 PMC12590094

[22] Nuttall T Mueller R Bensignor E Verde M Noli C Schmidt V . Efficacy of a 0.0584% hydrocortisone aceponate spray in the management of canine atopic dermatitis: a randomised, double blind, placebo-controlled trial. Vet Dermatol. (2009) 20:191–8. doi: 10.1111/j.1365-3164.2009.00756.x 19374721

[23] Rigaut D Briantais P Jasmin P Bidaud A. Efficacy and safety of a hydrocortisone aceponate-containing ear spray solution in dogs with erythemato-ceruminous otitis externa: a randomised, multicentric, single-blinded, controlled trial. Vet Dermatol. (2024) 35:197–206. doi: 10.1111/vde.13224 38093088

