# Supplementary material for: Randomized comparative field clinical trial of hydrocortisone aceponate/gentamicin/miconazole otic suspension vs. mometasone furoate/florfenicol/terbinafine otic solution for the treatment of canine otitis externa
Source: Front Vet Sci. 2026 Oct 28;13:1901247. doi: 10.3389/fvets.2026.1901247 (PMC13755693; doi:10.3389/fvets.2026.1901247)
Supplement: Supplementary file 1 [file Data_Sheet_1.pdf]

Supplementary Material 1 : distribution of enrolled dogs

	Easotic group (N=43)	Easotic group (%)	Claro group (N=43)	Claro group (%)
Sex				
Male	18	42	29	67
Intact male	4	9.3	7	16
Neutered male	14	33	22	51
Female	25	58	14	33
Intact female	6	14	2	4.7
Neutered female	19	44	12	28
Selected ear				
Left ear	20	47	17	40
Right ear	23	53	26	60
Age (years)				
Mean	5.0		5.5	
Min	0.7		0.7	
Max	16.1		12.0	
Body Weight (kg)				
Mean	27		27	
Min	4		3	
Max	59		51	
Baseline OTIS3 score				
Mean	7.56		7.65	
Min	5		5	
Max	11		11	
Baseline cytological score				
Mean	4.93		4.98	
Min	1		3	
Max	8		11	
