# Supplementary material for: Randomized comparative field clinical trial of hydrocortisone aceponate/gentamicin/miconazole otic suspension vs. mometasone furoate/florfenicol/terbinafine otic solution for the treatment of canine otitis externa
Source: Front Vet Sci. 2026 Oct 28;13:1901247. doi: 10.3389/fvets.2026.1901247 (PMC13755693; doi:10.3389/fvets.2026.1901247)
Supplement: Supplementary file 2 [file Data_Sheet_2.pdf]

Supplementary Material 2 : distribution by study site

	SITE 1	SITE 2	SITE 3	SITE 4	SITE 5	SITE 6	SITE 7
**Number of dogs included**	**13**	**10**	**1**	**29**	**16**	**14**	**3**
Easotic-treated group	7	5	0	15	8	7	1
Claro-treated group	6	5	1	14	8	7	2
Left ear	6	1	0	14	8	7	1
Right ear	7	9	1	15	8	7	2
